# Signal extraction from movies of honeybee brain activity: the ImageBee plugin for KNIME

**DOI:** 10.1186/1471-2105-14-S18-S4

**Published:** 2013-11-05

**Authors:** Martin Strauch, Julia Rein, Christian Lutz, C Giovanni Galizia

**Affiliations:** 1 Bioinformatics and Information Mining, University of Konstanz, 78457 Konstanz, Germany; 2 Neurobiology, University of Konstanz, 78457 Konstanz, Germany

## Abstract

**Background:**

In the antennal lobe, a dedicated olfactory center of the honeybee brain, odours are encoded as activity patterns of coding units, the so-called glomeruli. Optical imaging with calcium-sensitive dyes allows us to record these activity patterns and to gain insight into olfactory information processing in the brain.

**Method:**

We introduce ImageBee, a plugin for the data analysis platform KNIME. ImageBee provides a variety of tools for processing optical imaging data. The main algorithm behind ImageBee is a matrix factorisation approach. Motivated by a data-specific, non-negative mixture model, the algorithm aims to select the generating extreme vectors of a convex cone that contains the data. It approximates the movie matrix by non-negative combinations of the extreme vectors. These correspond to pure glomerular signals that are not mixed with neighbour signals.

**Results:**

Evaluation shows that the proposed algorithm can identify the relevant biological signals on imaging data from the honeybee AL, as well as it can recover implanted source signals from artificial data.

**Conclusions:**

ImageBee enables automated data processing and visualisation for optical imaging data from the insect AL. The modular implementation for KNIME offers a flexible platform for data analysis projects, where modules can be rearranged or added depending on the particular application.

**Availability:**

ImageBee can be installed via the KNIME update service. Installation instructions are available at http://tech.knime.org/imagebee-analysing-imaging-data-from-the-honeybee-brain.

## Introduction

### Biological background

**Figure 1:**
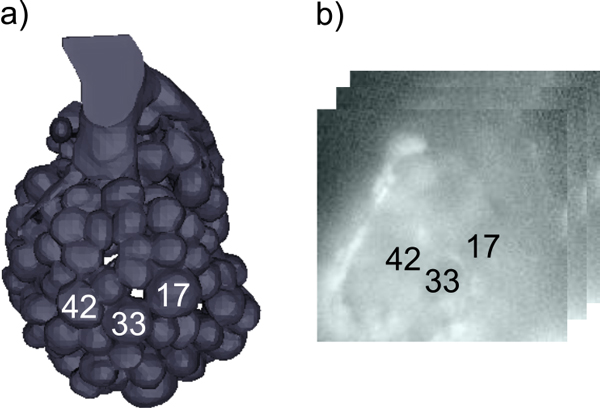
**Imaging the AL**. a) Anatomical model of the honeybee AL (generated from the average-shape atlas [31]), illustrating the view on the AL in calcium imaging experiments. Landmark glomeruli are labelled according to [30]. b) Schematic for a calcium imaging movie. The image on top of the stack is the ratio of images recorded with excitation light at wavelengths 340 nm and 380 nm, which is the input signal for the algorithm. For orientation, positions of the landmark glomeruli are marked by glomerulus numbers/labels. Constructing glomerular maps, as with the algorithm described in this work, facilitates identification of the glomeruli.

Optical recordings with calcium-sensitive, fluorescent dyes can be used to measure insect brain activity. In particular, we address questions regarding the olfactory system of honeybees. Across organisms, odours are encoded by activity patterns [1] in dedicated brain regions, such as the olfactory bulb in mammals, or the insect antennal lobe (AL). The AL of the honeybee *Apis mellifera *provides a 160-dimensional coding space, where a particular odour elicits a characteristic response pattern across the 160 glomeruli, the functional units of the AL [2]. Figure 1a shows an anatomical model of the honeybee AL.

Recording many odour response patterns gives rise to the species' olfactome, the entire odour coding space that defines odour similarity and dissimilarity according to the species' sensory input (see e.g. [3] for the *Drosophila *olfactome). Apart from recording odour response patterns, observing the honeybee AL allows us to answer questions about odour learning and memory. Honeybees can be conditioned to associate an odour with a (sugar) reward, and this can lead to changes in the odour response pattern after learning the association with the reward [4]. Even in the absence of odour stimulation, reverberations of past odour response patterns can be detected [5], suggesting a role in short-term memory.

Glomerular odour response patterns can be recorded by imaging with calcium-sensitive fluorescent dyes [5][6][4]. For this work, we used the dye Fura2-dextran to record from the honeybee AL. After exciting the dye with light at a frequencies 340 nm and 380 nm, a CCD camera on top of a confocal microscope recorded sample fluorescence. Fluorescence changes are proportional to the changes in intracellular calcium, a proxy signal for neuron firing rate [7]. For an example, see Figure 1b: The input for data processing consists of calcium imaging movies of brain activity recorded with a temporal resolution of 4-5 Hz. In the movies, a subset of the 160 glomeruli (see Figure 1a) can be observed.

During the experiments used for this work, several movies recorded from the same bee were concatentated. Movies were either recordings of odour responses or recordings of periods without odour stimulation. As glomeruli have characteristic odour responses, stimulation with different odours decorrelates the glomerular signals. As glomeruli also have individual spontaneous background activity in the idle state, also recordings without odour stimulation contribute to decorrelating glomerular signals. Correlations between pixels (time series) from the same glomerulus, and the fact that pixels from different glomeruli are (in approximation; for long recordings) uncorrelated, is the basis for detecting glomerular signals and for extracting them from the imaging movies.

### Motivation and outline

Imaging with calcium-sensitive dyes allows us to record the signals of many glomeruli simultaneously, gaining access to the glomerular odour response patterns. Before evaluating the response patterns, the challenge for data analysis is to detect glomerulus positions in the movies and to accurately estimate their signals.

In order to provide a solid foundation for data analysis and to enable automatic processing of imaging movies, we have developed a plugin, ImageBee, for the data analysis framework KNIME [8]. The graphical user interface of KNIME facilitates construction of data pipelines or workflows, where algorithms are embedded in a chain of pre- and postprocessing steps. Each step is implemented by a module ("node") in the KNIME workflow. ImageBee is a flexible platform for the analysis of imaging data from the honeybee brain. Due to the modular principle of KNIME, it can be easily extended or adapted to new kinds of data from emerging technologies such as two-photon microsopy [9].

Analysis of calcium imaging movies has traditionally been performed semi-automatically, using software to preprocess data, to visualise correlations between time series, and then to select regions of interest, that correspond to the glomeruli, by visual inspection [10][6]. In contrast, we present an algorithm, along with an implementation for KNIME/ImageBee, that automatically extracts glomerular signals from calcium imaging movies of the honeybee AL.

Similar to concepts from remote sensing [11][12], an imaging movie can be described by a non-negative mixture model. Pixels (time series) are assumed to contain either pure glomerular signals, or, in regions of contact, the additive mixture of one or more glomerulus signals. This is motivation for a convex analysis approach that aims to select the generating extreme vectors of a convex cone containing the data. These correspond to the pure glomerulus signals that can be combined, with non-negative coefficients, to reconstruct also the mixtures.

In the following, we first review related work and then introduce the algorithm (Methods), followed by a description of the ImageBee plugin, and evaluation of the algorithm on biological, as well as on artificial data (Results and Discussion).

### Relationship to the conference version

This article is an extended journal version of the conference paper that appeared in the proceedings of ICCABS 2012 [13]. In the conference version, we developed the main algorithm for processing imaging movies. The journal version introduces the ImageBee plugin for KNIME that contains implementations of the methods described in this work, as well as a variety of tools for handling, processing and visualising imaging data. Furthermore, the journal version provides additional details on the algorithm and points out relationships to other methods.

### Related work

Data analysis for calcium imaging movies is still dominated by semi-manual methods that involve user interaction to identify glomerulus positions (e.g. [5], [6], [4]).

Algorithmic solutions can be classified as synthetic or analytic. In a synthetic approach, Stetter et al. [14] defined nonlinear model functions, e.g. functions describing odour response and dye bleaching. They reconstructed honeybee imaging data by estimating the contribution of each of the model functions to time series from the movie. This approach does, however, not separate glomeruli.

Analytic approaches attempt to decompose the movie matrix into components that correspond to signals or latent factors underlying the data. Reidl et al. [15] and Strauch&Galizia [16,17] applied Independent Component Analysis (ICA) to imaging movies. While ICA is able to decompose imaging movies into glomerular signals, these approaches are adaptations of a general paradigm to imaging data. They rely on statistical model assumptions - independence and non-Gaussianity for all but one of the source signals, and do not consider non-negativity.

In contrast, we propose a data-specific mixture model that avoids these strict statistical model assumptions and incorporates a non-negativity constraint that proves beneficial with respect to interpretability of the factors. Based on the non-negative mixture model, we then develop an algorithm that identifies the pure glomerular signals as the extreme vectors of a convex cone that contains the data (see Methods).

The convex analysis approach has previously found application in remote sensing and the analysis of hyperspectral data [18]. In particular, Ifarraguerri&Chang [11] and Gruninger et al. [12] have proposed related algorithms that also aim at finding a convex cone containing the data. For remote sensing applications, the goal is to identify so-called endmembers, the signals of pure materials or soil types, that can be used to unmix the signal of a given pixel, decomposing it into contributions by the pure materials. Our algorithm utilises a convex cone approach to find and to select the pure glomerular signals, which are then postprocessed to remove residual noise. Mixed-signal pixels are not unmixed, but discarded, as only the pure glomerular signals are relevant features for the analysis of glomerular odour response patterns. Another strategy to distinguish glomeruli based on their differential activity would be to select pixels that are cluster centers. However, clustering algorithms may group pure and mixed-signal pixels from the same glomerulus into one cluster, or they may result in overclustering, creating additional clusters for the signal mixtures. Employing the mixture model and the convex cone approach is what renders our algorithm robust against selecting mixed-signal features.

Our algorithm can be seen as a feature selection approach that selects a subset of pixels (corresponding to the extreme column vectors). The approach is, however, unsupervised, and the term "feature selection" is often used in a supervised context, where features are sought that improve classification success [19]. Our algorithm utilises signals selected from the movie matrix as basis vectors in a matrix factorisation framework. In this respect, it can be understood as performing a column-based matrix factorisation of the kind *A *≈ *CX*, where the *m *× *n *matrix *A *is approximated with a subset of its columns in *C *(*m *× *c*) that are combined by (non-negative) coefficients in *X *(*c *× *n*). Column-based matrix factorisation approaches [20,21] focus on *selecting *the most relevant vectors from a matrix to use them as interpretable basis vectors, as opposed to *generating *features by linear combination, such as in PCA and ICA.

## Methods

### Matrix factorisation framework for imaging movies

An imaging movie can be represented as a *m *× *n *matrix *A*, where *m *is the number of time points and *n *the number of pixels. The movies used for evaluation in this paper have 140 × 130 pixels and about 4,000 time points, where both numbers could be higher in theory. They depend on the chosen resolution of the recording and e.g. on the number of odour stimulations.

We can factorise *A *as follows, using only *k *factors, giving rise to the approximated *A_k_*:

(1) $$A^{m\;\times \;n}:A_{k}=T^{m\;\times \;k}S^{k\;\times \;n}=\overset{k}{\underset{r=1}{\sum }}T_{Ir}\;S_{rJ}$$

While being an approximation, *A_k_*should still be similar to the original matrix *A *in the sense that the Frobenius norm error $∥A-A_{k}{∥}_{Fr}$ is small. In Equation 1, matrix *T *has a temporal interpretation as it contains *k *time series, and matrix *S *has a spatial interpretation as it contains *k *images.

Regarding notation, *A_Ij_*is the *j*th column of *A*, i.e. the *j*th pixel or pixel time series. The rows of *S *are denoted as **s**_(*r*) _and the columns of *T *as **t**^(*r*)^.

With respect to minimising $∥A-A_{k}{∥}_{Fr}$, the optimal solution is given by the principal components of *A*. For the sake of interpretability, we will introduce further constraints and demand that *T *should be restricted to columns selected from *A *(or at least to sparse combinations, i.e. averages over a limited number of similar vectors), and that *S *should be non-negative. The ideal factorisation should yield the glomerular signals in the columns of *T*, and images describing the position of the glomeruli in the rows of *S*. As we will deal with both cases, a principal component solution, and a column-based solution, we reserve *k *for the number of principal components and denote the principal component solution as *A_k_*. We use *c *to refer to the number of columns selected from *A*, and *A_c_*for the column-based solution.

In the following, we describe an algorithm for computing a matrix factorisation with the desired properties. The algorithm can be structured into three main steps:

1. Preprocessing by z-score normalisation and PCA.

2. Convex cone fitting (Algorithm 1).

3. Postprocessing to remove residual noise.

Depending on the nature of the data, additional preprocessing steps may be added, such as spatial filtering of the images or correction for animal movement.

### Preprocessing with PCA

For each *A_Ij_*, we computed the mean *µ_j_*and the standard deviation *σ_j_*, and then performed z-score normalisation as *A_Ij_*:= (*A_Ij_*− *µ_j_*)/*σ_j_*.

We then performed Principal Component Analysis (PCA) [22], computing the top-*k *principal components of *A*. In the notation from Equation (1), matrix *S *contains the *k *principal component images, and the corresponding loadings are in matrix *T*. Similarly, computing PCA on *A^T ^*would give rise to principal component time series.

**Figure 2:**
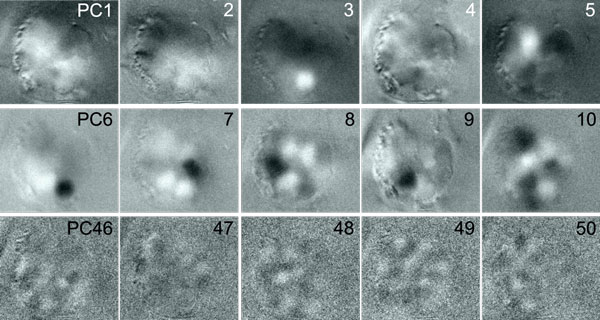
**Principal Components of an Imaging Movie**. Selected principal component images of a calcium imaging movie. Principal components are sorted by eigenvalue (highest to lowest). For each image, the colour scale is min-max, i.e. white corresponds to negative values, and black to positive values.

Fixing *k *and denoting as *P_k_*the matrix of the top-*k *principal components, PCA minimises $∥P_{k}^{T}P_{k}-A^{T}A{∥}_{Fr}$( [22], chap. 3.2). Thus, PCA preserves the covariance structure in the movie, such as the covariance between pixels that belong to the same glomerulus. Optimal preservation of the covariance structure and optimal reduction of the Frobenius norm error ensure a good approximation to *A *by PCA. Dimensionality reduction by PCA allows any subsequent algorithm to be carried out on a much smaller matrix. The principal component images in *S *(Figure 2) illustrate another important aspect of PCA: Signals are accumulated in the top principal components. The (first) principal component is the variance-maximising projection [22]. Transient signals, such as glomerular odour responses or spontaneous activity, contribute to the variance. Glomerular signals are thus concentrated in the top principal components with high eigenvalues, allowing to discard components with lower eigenvalues that contain mostly noise (Figure 2).

Both preprocessing steps, z-score normalisation and PCA, help to increase the prominence of glomerular signals in the movies. Due to its optimality properties, PCA is of general value. Z-score normalisation proved beneficial on the Fura2-dextran recordings used in this work, but it may not be as useful for recordings with other dyes that exhibit stronger bleaching when exposed to light.

### Convex cone fitting

The movie matrix *A *can be described by the following data model:

(2) $$A=TS^{0+}+N$$

A set of time series in the columns of *T *can be combined with non-negative coefficients in *S*^0+ ^to reconstruct *A *up to the residual noise *N*. Each time series from *A *is either represented by one of the basis time series in *T*, or it can be modelled as a combination of several time series in *T *with non-negative coefficients. The model assumption is that we can observe pure signal sources in the middle of the glomeruli, whereas at the fringes of the glomeruli light scatter from neighbouring glomeruli can lead to *additive *signal mixtures. Ideally, the pure signal sources should be selected into *T*, and mixtures can then be modelled with the help of the coefficients in *S*^0+^.

For the moment, we omit the noise term *N*, which is dealt with in postprocessing (see next section). We assume that the pure signal sources are present in *A*, and we will use concepts from convex analysis to find them.

We first introduce definitions: A set of vectors *V *is called a convex cone if *α*_1_**v**_1 _+ *α*_2_**v**_2 _∈ *V*, where *α*_1_, *α*_2 _are non-negative and **v**_1_, **v**_2 _∈ *V*. Linear combinations with non-negative coefficients are also called "conic combinations". By definition, the extreme vectors of a convex cone are those that cannot be reconstructed by conic combination. However, perfect reconstruction of all other vectors in *V *is possible by conic combination of the extreme vectors[23][24].

Returning to Equation 2, the columns of matrix *T *span a convex cone that is pointed at the origin. The cone encloses a part of *A *that can be reconstructed perfectly by conic combination of the columns of *T*, and the remaining data points are reconstructed (with an error) by projection to the closest point on the boundary of the cone. Thus, if we choose the set of extreme vectors of *A *into *T*, the cone defined by *T *encloses all data points and we achieve perfect reconstruction. Interestingly, the extreme vectors do not only guarantee us perfect reconstruction, but they also identify the pure signal sources, as, by the definition, the extreme vectors cannot be reconstructed by conic combination.

We thus propose Cone_fitting (Algorithm 1) as a heuristic to find extreme vectors. The strategy is a greedy forward heuristic, selecting at each iteration the column that is least explained by conic combination of the columns selected so far. Thereby, we make a locally optimal choice with respect to selecting an extreme vector.

During *c *iterations, *r *= 0, . . . , *c *− 1 columns **t**^(*r*) ^are selected into *T*, and the current version of matrix *A, A*^{*r*}^, is downdated by removing the influence of **t**^(*r*)^. In particular, we compute the corresponding spatial mapping **s**_(*r*) _= *A^T^***t**^(*r*) ^and then compute ${\text{s}}_{\left(r\right)}^{0+}$ by projecting negative entries in **s**_(*r*) _to zero. Then, we downdate *A *by setting $A^{\left\{r+1\right\}}=A^{\left\{r\right\}}-{\text{t}}^{\left(r\right)}{\text{s}}_{\left(r\right)}^{0+}$. The next column is chosen as the column with the highest Euclidean norm in *A*^{*r*+1}^. This stepwise removal of the chosen column, and everything that can explained by it with conic combinations, works towards selecting a non-redundant set of vectors. As the iteration proceeds, the norm of mixed signal columns is reduced by the downdating, rendering them less likely to stand out as the highest norm column that is the candidate for the next extreme vector. The first vector **t**^(0) ^needs to be initialised. For example, we could choose the vector with the largest Euclidean norm in *A*. For this work, we chose the column with the largest distance to a randomly selected column in order to obtain a vector from the hull, more explicitly avoiding to start the iteration with a mixed-signal column.

**Figure 3:**
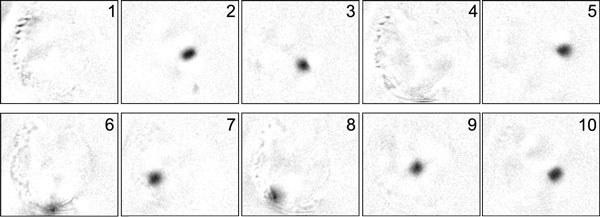
**Applying Algorithm 1 to a calcium imaging movie (same movie as in Figure 2): The images show the top-10 rows of matrix S**.

**Figure 4:**
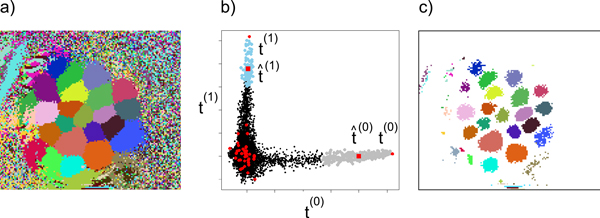
**Clustering and Postprocessing**. **a) **Induced clustering, where each pixel is assigned to the row of *S *(see Figure 3) where it has the highest coefficient. **b) **Visualisation of the signal refinement procedure in postprocessing. The movie matrix is projected onto the top-two signals, **t**^(0)^, **t**^(1) ^∈ *T*. Every black dot corresponds to a time series (pixel), and all time series that have been selected into *T *are shown as red dots. After averaging over the time series in the coloured areas, signals are shifted from **t**^(0) ^to the mean ${\hat{t}}^{\left(0\right)}$ and from **t**^(1) ^to the mean ${\hat{t}}^{\left(1\right)}$**c) **Glomerular map: Induced clustering based on  Ŝ, i.e. after postprocessing. Pixels that do not participate in any mean signal are set to white.

Figure 3 visualises the results of applying Algorithm 1 to the calcium imaging movie from the PCA example (Figure 2). The images in Figure 3 are visualisations of the row vectors of matrix *S*. They contain the spatial mapping of the selected (glomerular) time series and indicate the position of the respective glomeruli (or other signal sources) in the image plane. Summarising matrix *S*, we compute the clustering induced by *S*: Figure 4a. Here, each pixel is assigned a colour depending on in which row (image) of *S *it has the highest coefficient. The induced clustering shows that clusters of pixels with similar signals exist, the glomeruli.

### Postprocessing to remove N

So far, we have not yet dealt with the noise term *N *(Equation 2). Furthermore, the induced clustering does not yet distinguish pure signals from mixed signals in regions of contact with other glomeruli. In Figure 4a, mixed signal pixels are discretised to the source signal with the strongest contribution. We employ a postprocessing step (Figure 4b) to remove the residual noise, as well as the mixed signals. This

**Algorithm 1 **[*T, S*]= Cone_fitting (*A*^(*m *× *n*)^, *c*)

    **for ***r *= 0 **to ***c *− 1 **do**

        **if **(*r *== 0) **then**

            initialisation of *p*: see main text

        **end if**

        ${\text{t}}^{\left(r\right)}=A_{Ip}^{\left\{r\right\}}/∥A_{Ip}^{\left\{r\right\}}∥$

        $s_{\left(r\right)}={A^{\left\{r\right\}}}^{T}\;{\text{t}}^{\left(r\right)}$

        $s_{\left(r\right)}^{0+}=\text{negative}\text{\_}\text{to}\text{\_}\text{zero}\left(s_{\left(r\right)}\right)$

        $T_{Ir}=t^{\left(r\right)};\;S_{rJ}=s_{\left(r\right)}^{0+}$

        $A^{\left\{r+1\right\}}=A^{\left\{r\right\}}-{\text{t}}^{\left(r\right)}{\text{s}}_{\left(r\right)}^{0+}$ //form residual matrix

        $p=\underset{p}{\text{argmax}}||{\text{A}}_{Ip}^{\left\{r+1\right\}}||$ //index of next column

    **end for**

leads to refined signals, and, with respect to the induced clustering, to a map of pure glomerular signals ("glomerular map", Figure 4c).

Algorithm 1 selects time series from the movie matrix, that are (in approximation) pure source signals and not mixed with other source signals. The residual noise *N *can be averaged out. The more time series from the same glomerulus we can use for averaging, the better. However, we should avoid to include time series that are more similar to another source signal.

We project *A *onto *T *and then average over all time series that are closer to a given **t**^(*r*) ^∈ *T *than to any other column of *T*. This is visualised in Figure 4b: *A *is projected onto the first two column vectors **t**^(0) ^and **t**^(1) ^∈ *T*. Each dot corresponds to one time series, where **t**^(0) ^and **t**^(1) ^end up at the extremes of the two "arms". The postprocessing step consists of averaging over the coloured dots, those that are closer to **t**^(0) ^or **t**^(1)^, respectively, than to any of the other **t**^(*r*) ^∈ *T*. The averaged signals are denoted as ${\hat{t}}^{\left(0\right)}$ and ${\hat{t}}^{\left(1\right)}$, respectively.

For the row vectors ${\hat{s}}_{\left(r\right)}$ (images), this means that all pixel coefficients are set to zero that do not contribute to the average ${\hat{t}}^{\left(r\right)}$. The effects of the postprocessing step can then be visualised by the new induced clustering in Figure 4c. White pixels do not participate in any average: They contain mixed signals that are not close enough to any of the pure signal sources. The coloured areas indicate the pixels which contribute to the average signal of the respective glomerulus. Spatially contiguous clusters appear as a property of the data: Neighbouring pixels from the same glomerulus are correlated over time. However, at no point does spatial contiguity enter as a criterion into the algorithm.

### Comparison to other methods

Manual approaches to process imaging data (see e.g. [10]) rely on the evaluation of correlations between time series: Correlations between neighbouring pixels (time series) are visualised, and then feature selection is performed manually after inspection of these visualisations. Our method automatically extracts pure signal vectors, that are typically found in the middle of the glomeruli, and the corresponding images in matrix *X *(Figure 3) can be interpreted as the spatial distribution of correlation with the basis signals in *T*. While PCA preserves the correlation structure in the data, different glomerular signals are not split up into different principal components, as it is accomplished by Algorithm 1. When using PCA to factorise the movie *A*, the images in *S *(Figure 2) are dense, with many non-zero pixels, whereas the convex cone fitting in Algorithm 1 results in sparse images with only few non-zero pixels (Figure 3). This illustrates the different concepts behind the two approaches: Principal components are means (in a subspace), whereas we select extreme vectors. While PCA is optimal with respect to reducing the Frobenius norm error, it can be argued that the extreme vector solution is more interpretable: The time series vectors corresponding to the images in Figure 3 can be interpreted as the signals of individual glomeruli, whereas this is not the case for the PCA solution (Figure 2).

### Implementation for KNIME

The central part of our method is the Cone_fitting procedure (Algorithm 1) that selects glomerular time series from an imaging movie. Yet, image analysis often requires that data be channelled through a pipeline of consecutive processing steps. In this work, we employ z-score normalisation as a preprocessing step and PCA for denoising and dimensionality reduction before performing Cone_fitting. Then, different steps may be taken: The user might wish to view a glomerulus map, the glomerular time series or a low-rank reconstruction of the movie matrix. Ultimately, novel methods might be developed that improve a certain part of the pipeline, or a preprocessing step that proved beneficial for the data at hand might not be as useful for other data types, e.g. when a different recording technique is employed.

**Figure 5:**
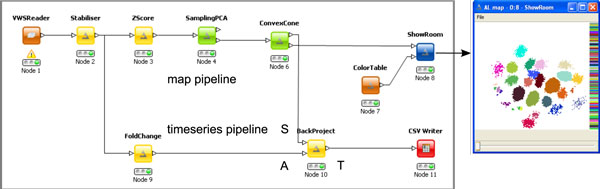
**KNIME Pipeline**. Image processing with the ImageBee plugin for KNIME. Movies are read in by the VWSReader node and corrected for animal movement with the Stabiliser node. The upper pipeline then follows the method described in this paper and computes a glomerular map that is displayed by the visualisation node ShowRoom. The lower pipeline normalises movies by subtracting the mean activity before odour stimulation (node FoldChange) and computes glomerular time series in matrix *T *given matrix *S *from the ConvexCone node (Algorithm 1) and the movie *A*. Time series are written to a CSV file. See main text for details on the individual processing steps.

Providing a user interface that is as flexible as it is easy to operate, we implemented our method for the open-access platform KNIME [8]. In a KNIME workflow, data processing pipelines can be arranged based on a modular principle where individual processing steps, such as PCA, are available as a nodes. Each node contains a Java program and individual nodes can be connected to create a data pipeline (Figure 5). For implementation, we relied on the libraries provided by KNIME, as well as on the Image Processing Plugin (http://tech.knime.org/community/image-processing) for handling image data within KNIME. Matrix operations were implemented using the CERN Colt (http://acs.lbl.gov/software/colt/) and Parallel Colt [25] libraries.

We implemented KNIME nodes for all the preprocessing steps described in this paper and the Cone_fitting algorithm, along with several other nodes for data visualisation, e.g. for creating a low-rank reconstruction of the original movie matrix and for visualising it using a false-colour scale. The modular architecture of KNIME ensures that the toolkit can be easily extended.

At any step in the pipeline, data can be written to files or taken over by other nodes available for KNIME that also features connections to ImageJ [26] and R [27]. Fixed processing pipelines can be summarised in meta nodes and loop nodes allow for repeated execution of the pipeline, e.g. by iterating over a list of files. We have bundled our KNIME nodes in a plugin, ImageBee, that is available online. ImageBee extends KNIME by the following nodes (details are available on the node description pages within KNIME).

• VWSReader: Reader for the TILL Vision format.

• Stabiliser: Correction for animal movement/shifts between movies by cross correlation.

• Normalisation: Zscore, FoldChange, HistogramNormalisation, ImageArithmetic: subtract/add other movies, e.g. a control measurement

• Filters: SpatialFilter, TemporalFilter

• PCA: SamplingPCA (exact PCA or approximate PCA by pixel sampling [17]), CCIPCA (incremental PCA [28])

• ConvexCone: Algorithm 1 from this paper

• Visualisation: ColorTable, ApplyColorTable, ShowRoom (viewer), OverlayMap (superimposes a glomerulus map onto movie)

• Matrix operations: MatrixMultiplication, BackProject (*T *:= *AS*), TimeseriesProjector (*S *:= *T *^−1^*A*)

• Helper nodes: ImageExtractor, SplitMovies, MergeMovies

### Example Pipeline in KNIME

For illustration of the data pipelining concept, refer to Figure 5, where we show a KNIME pipeline implementing the method described in this paper. Imaging movies are read by a reader node (TILL Vision container format ".vws"). As the data is a concatentation of movies recorded intermittently in the same bee (Figure 1b), the individual movies might be slightly shifted due to animal movement or changes in experimental setup. If necessary, the Stabiliser node aligns subsequent movies optimally by cross correlation.

Then, the data flow is split into two pipelines, where the upper pipeline computes a glomerular map and the lower pipeline computes glomerular time series. For computing time series, the movie is only treated with FoldChange that is configured to normalise time series by subtracting the mean during the interval before odour stimulation, thus highlighting changes due to the stimulation.

As Algorithm 1 is performed in PCA space where the time dimension is reduced to *k*, the output of ConvexCone is the spatial matrix *S *(see Figure 3), and not the time series matrix *T *. For the KNIME implementation (Figure 5) we thus employ the node BackProject that computes the full-length matrix *T *by projecting the movie matrix *A *onto *S*. Time series are then written to a text file for further analysis, e.g. with a statistics software. By default, ConvexCone performs signal refinement to remove residual noise, i.e. the procedure from the KNIME pipeline in Figure 5 is equivalent to computing matrices $\hat{T}$ and  Ŝ (Figure 4).

In the upper pipeline, a glomerular map is constructed as described above by first z-score normalising the movie (Zscore), applying PCA and Algorithm 1 (ConvexCone). Here, the SamplingPCA node is configured to compute exact PCA. Approximations to PCA that can lead to considerable speedups are discussed below.

### Availability

The ImageBee plugin for KNIME (http://www.knime.org) is available online via the update site for KNIME community contributions: http://tech.knime.org/update/community-contributions/nightly. Please install ImageBee and the KNIME Image Processing plugin (required for handling image data in KNIME). Detailed installation instructions, downloadable KNIME pipelines (for glomerular maps, time series, low-rank reconstructed movies), and an example dataset are available at http://tech.knime.org/imagebee-analysing-imaging-data-from-the-honeybee-brain.

## Results and discussion

### Computational complexity

Computational complexity of Algorithm 1 is dominated by forming the *m *× *n *residual matrix *c *times. After dimensionality reduction with PCA, the computational effort for performing Algorithm 1 on a small matrix is negligible. The computational load of the entire method depends mainly on preprocessing, and in particular on PCA.

In our KNIME plugin, we provide three algorithms for computing PCA. The SamplingPCA node implements an iterative PCA approach (NIPALS, [29]).

Optionally, a speedup can be achieved by pixel importance sampling. For example, by sampling 5% of the pixels based on a biologically motivated importance criterion [17], running time can be reduced to about 5% of the running time for exact PCA. We have previously shown [17] that PCA with pixel sampling leads to high quality approximations to the principal components on imaging data.

Another variant is an incremental PCA approach, CCIPCA [28]. The CCIPCA algorithm finds an approximate solution to PCA by incrementally processing a movie stream. It is a memory-efficient way of computing PCA, as, at any given time point, only the current image from the movie stream and the principal components are kept in memory.

Iterative PCA has a complexity of O(mnki) where *m *and *n *are the movie dimensions, *k *is the number of principal components and *i *the number of iterations until convergence. The pixel sampling approach can substantially decrease the number of pixels *n *and thereby complexity of iterative PCA. Incremental PCA with the CCIPCA algorithm does not reduce *n*, but it saves the factor *i *as no convergence is involved, leading to a complexity of O(mnk)[28].

### Parameter settings

Two parameters need to be set, the number of principal components *k *and the number of time series/basis signals *c *for Cone_fitting (Algorithm 1). In both cases, default values (k = c ≈ 50) can be employed for typical calcium imaging recordings of the honeybee AL (as used in this work or e.g. in [4,10]).

For very large *k *we would sacrifice the dimensionality reduction aspect of PCA, while information would be lost by choosing a very small *k*. From the typical dataset in Figure 2 it is apparent that values of *k *around 50 are a good compromise in this respect.

Regarding *c *it should be noted that Cone_fitting returns nested basis vectors, i.e. given a fixed starting point, the *n*th basis vector remains the same, regardless of the size of *c*. This is different from a common behaviour for clustering algorithms where cluster centers move when the number of clusters is changed. As we know that there are between 20 and 40 glomeruli that can be visible at the most (see model in Figure 1a), setting *c *to a value in this range is a reasonable choice. Choosing a slightly larger *c *accounts for the presence of non-glomerular objects in the movies that also have their own signals.

While, in principle, *c *is an uncritical parameter, it can become relevant for signal refinement in postprocessing (Figure 4). For large *c*, the distance to the closest basis signal tends to decrease, simply because there are more basis signals. This leads to averages over a smaller amount of time series (coloured dots in Figure 4b) and thus to smaller clusters (Figure 4c). Thus, especially for recordings with a small number of glomeruli it can be helpful to lower *c *in order to increase cluster size.

### Evaluation on artificial data

For evaluation, we constructed artificial datasets with 16 known source signals each. The biological datasets used in this work contain both recordings of odour responses and of spontaneous background activity in the idle state. Generally, odour response recordings have high peak amplitudes directly after odour presentation, while spontaneous activity has lower amplitudes.

**Figure 6:**
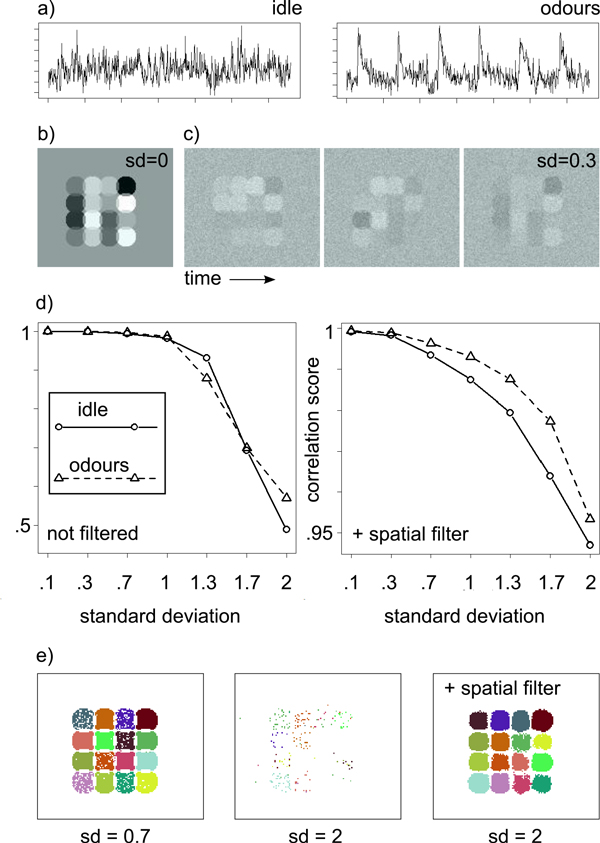
**Evaluation: Artifical Data**. **a) **Example sources for the "idle" and "odours" datasets. **b) **Image from an artificial imaging movie (no noise added). Artificial glomeruli are circular and overlap. **c) **Consecutive images from an artificial imaging movie (noise with *σ *= 0.3 added) **d) **Correlation scores for varying noise levels on the "idle" and "odours" datasets. Left: Correlations scores for data without smoothing by spatial filtering. Right: Smoothing with a Gauss filter improves correlation scores for high noise levels. **e) **Glomerular map for the "idle" dataset. From left to right: Noise level *σ *= 0.7, noise level *σ *= 2 without spatial filtering, noise level *σ *= 2 with additional spatial filtering.

To check for possible performance differences between the data types, we used two artificial datasets, "odours" and "idle". For both datasets, source signals were derived from real measurements (see Figure 6a for examples), i.e. signals were distinct, but not perfectly uncorrelated. For both artificial datasets, source signals (*µ *= 0*, σ *= 1, shifted to be non-negative) were assigned to spatially contiguous, partially overlapping clusters (Figure 6b). In regions of overlap, signals were additive mixtures. Finally, Gaussian noise (with standard deviation *σ*) was added (Figure 6c).

We then measured how well the implanted source signals could be recovered from the artificial datasets for varying noise levels *σ*. Source recovery is expressed by a correlation score based on the Pearson correlation coefficient *ρ*(x, y) between time series vectors **x **and **y**. For a recovered signal ${\overset{⌢}{t}}^{\left(i\right)}$ and known source signal **u**^(*j*)^, the correlation score is defined as:

(3) $$\text{corr}=\frac{1}{n}\overset{n}{\underset{i=1}{\sum }}argmax_{j}\;\rho \left({\hat{t}}^{\left(i\right)},u^{\left(j\right)}\right)$$

We performed Algorithm 1 (with peprocessing by PCA) on both artificial datasets. Across a range of noise levels from *σ *= 0.1 to *σ *= 1, sources could be successfully recovered on both datasets (Figure 6d). A sharp decline in source recovery occurred for *σ > *1, which, however, could be remedied by spatial filtering. In order to reduce noise, we smoothed images with a Gaussian kernel (width = 7). With the additional spatial filtering, source recovery was possible even for higher noise levels up to *σ *= 2.

Figure 6e illustrates source recovery, showing the induced clusterings. For low noise, all sources are clearly visible as clusters, whereas mixed signals in regions of overlap are excluded and set to white. For higher noise levels, the sources could still be detected, however very few pixels were sufficiently close to the selected source signal. This left fewer pixels to average over in the postprocessing step, leading to low correlation scores. Noise reduction by spatial filtering rendered more pixels sufficiently close to the source signal, which resulted in smoother clusters and better source recovery due to averaging over a larger number of pixels.

Source recovery was similar on both data types. For high noise levels, in particular on spatially filtered data (Figure 6c), performance was slightly better for the "odours" dataset than it was for the "idle" dataset. This can be explained by the clearer signals during odour responses, as opposed to low amplitude fluctuations in spontaneous activity that are more susceptible to high noise levels.

### Evaluation on biological data

**Figure 7:**
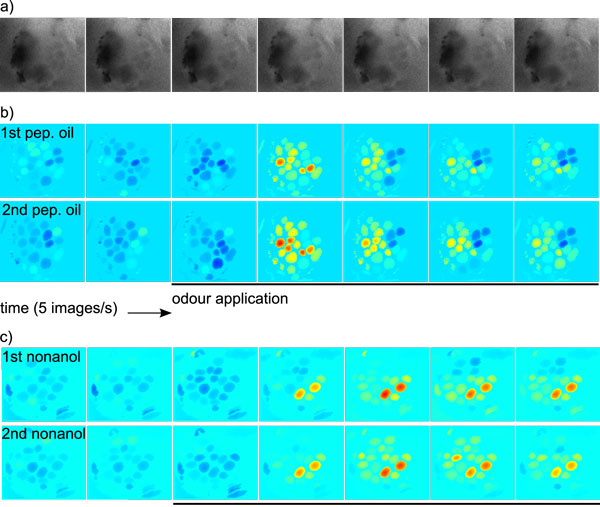
**Evaluation: Biological Data**. **a) **Consecutive images (340 nm/380 nm) from a calcium imaging movie of the honeybee AL. **b) **Low-rank reconstruction with the method presented in this work. During the interval marked with the black bar, the odour peppermint oil was administered to the bee. The two rows show two responses to the odour peppermint oil measured in the same bee. The images in the first row ("1st. pep. oil") correspond to the raw images in a). Each pixel has been normalised to its mean intensity before odour application. The colour scale is min-max (blue-red) for each row. **c) **In another bee, responses to the odour nonanol were measured. Data treatment and colour scale as in b).

Finally we tested the ImageBee plugin on imaging movies from the honeybee AL (Fura2-dextran stainings) that contained measurements of both odour responses and spontaneous background activity. For each imaging movie, we performed Algorithm 1 with PCA preprocessing and followed by spatial filtering. As a result, we obtained for each movie a time series matrix $\hat{T}$, an image matrix  Ŝ, and a glomerular map. Figure 7 shows low-rank reconstructions of imaging movies, computed as $A_{c}=\hat{T}Ŝ$ and displayed in a false-colour scale. This visualises the response pattern sequence after odour stimulation. For raw images, see Figure 7a. Low-rank reconstructed images are shown in Figure 7b (odour: peppermint oil) and Figure 7c (odour: nonanol). In both cases, the same odour response has been measured twice, giving an impression of inter-trial variability. While there is variability in the glomerular odour responses, both within and between animals, stable odour representations can be obtained by averaging over many responses. In fact, odour responses in the honeybee are sufficiently characteristic to speak of a species-specific olfactory code [2].

**Figure 8:**
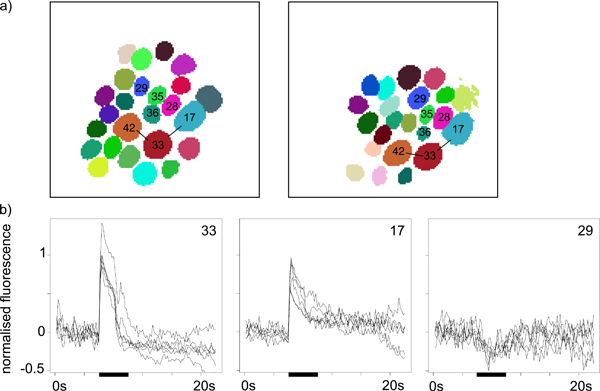
**Glomerular time series**. **a) **Glomerular maps for two different bees. Landmark glomeruli are labelled according the anatomical reference AL. Maps have been edited (to remove non-glomerular clusters) and labelled with ImageBee. **b) **Glomerular odour responses (time series from $\hat{T}$) for three different glomerulus types. Glomerulus labels as in a). Data is pooled from two odour presentations (nonanol) in each of three bees.

Based on the glomerular maps, we could determine the identity of landmark glomeruli using an anatomical model of the honeybee AL [30] (s.a. Figure 1). Figure 8a shows glomerular maps from two different bees. The number of glomeruli in a map depends on several experimental parameters, e.g. the focal plane of the recording. Despite experimental and also biological variation in individual bees, AL anatomy is partly conserved between bees (Figure 8a).

This allows for comparing odour response time series (matrix $\hat{T}$) for glomeruli that have been identified based on their position in the glomerular maps. Figure 8b shows time series for three different glomerulus types in response to the odour nonanol. Time series in Figure 8b are pooled data from several animals: Two odour responses were measured in each of three different bees. Despite some variability, response amplitude and temporal dynamics of the glomerular odour responses are conserved, both within and between animals.

### Conclusions

We have introduced the ImageBee plugin for KNIME as a platform for analysing and visualising imaging data from the insect AL. Previously, data analysis in this field required manual selection of regions of interest (as e.g. in [5][6][4]). The ImageBee plugin enables automatic detection of glomerulus positions in calcium imaging movies (Figure 8a), it automatically extracts glomerular time series (Figure 8b), and it can be used to produce denoised versions of the imaging movies by low-rank reconstruction (Figure 7). At the core of the image processing pipelines that can be constructed with ImageBee lies an algorithm (Algorithm 1) that is based on a data-specific mixture model and that leads to a factorisation of the movie matrix with interpretable basis vectors. These are either time series selected from the movie matrix, or, after postprocessing, combinations, with non-negative coefficients, of a limited number of similar time series vectors. This interpretability aspect is what distinguishes our method from more general approaches, such as PCA, where basis vectors can be linear combinations of a large number of vectors with mixed-sign coefficients.

While the software has been designed for and tested on honeybee recordings with Fura2-dextran, a large number of KNIME nodes for data pre- and postprocessing are available, such that it can be adapted to other kinds of data with similar properties, e.g. imaging movies from other insect species, recordings that rely on other calcium dyes or on other techniques, such as multi-photon microscopy. In all these cases, a so-called functional segmentation of the image plane can be achieved, a segmentation into units (glomeruli) with similar signals over time.

Reproducible results by automated and deterministic processing, accurate estimation of glomerular time series, along with visualisation of the spatial aspect of odour response patterns by denoised movies, are the basis for analysing data on the physiology of olfactory coding, odour learning and memory.

## Competing interests

The authors declare that they have no competing interests.

## Authors' contributions

MS developed methods and wrote the manuscript. MS and CL performed programming. JR performed biological experiments. CGG supervised research and edited the manuscript. All authors read and approved the final manuscript.

## Declarations

Publication of this article was supported by the University of Konstanz.

This article has been published as part of *BMC Bioinformatics *Volume 14 Supplement 18, 2013: Selected articles from the Second IEEE International Conference on Computational Advances in Bio and Medical Sciences (ICCABS 2012): Bioinformatics. The full contents of the supplement are available online at http://www.biomedcentral.com/bmcbioinformatics/supplements/14/S18.
